# History of research concerning the ependyma: a view from inside the human brain

**DOI:** 10.3389/fncel.2023.1320369

**Published:** 2024-01-08

**Authors:** Marc R. Del Bigio

**Affiliations:** Department of Pathology, Rady Faculty of Health Sciences, University of Manitoba, Winnipeg, MB, Canada

**Keywords:** brain, cerebrospinal fluid, cilia, cytology, ependyma, neuropathology, ventricular system

## Abstract

The history of research concerning ependymal cells is reviewed. Cilia were identified along the surface of the cerebral ventricles c1835. Numerous anatomical and histopathological studies in the late 1800’s showed irregularities in the ependymal surface that were thought to be indicative of specific pathologies such as syphilis; this was subsequently disproven. The evolution of thoughts about functions of cilia, the possible role of ependyma in the brain-cerebrospinal fluid barrier, and the relationship of ependyma to the subventricular zone germinal cells is discussed. How advances in light and electron microscopy and cell culture contributed to our understanding of the ependyma is described. Discoveries of the supraependymal serotoninergic axon network and supraependymal macrophages are recounted. Finally, the consequences of loss of ependymal cells from different regions of the central nervous system are considered.

## Introduction

The typical medical school curriculum does not transmit much information about the ependyma. There are perhaps two slides in an introductory neurocytology lecture and passing mention in lectures concerning neurodevelopment and cerebrospinal fluid (CSF) physiology. I started thinking about ependymal cells in 1983 when I began my PhD studies, investigating the pathogenesis of hydrocephalus and shunt obstruction. My mentor was Dr. J. Edward Bruni, a neuroanatomist and electron microscopist who had been studying tanycytes and their role in brain physiology (Bruni, 1974). Leap forward 40 years, in the course of studying over 6,000 human brains in detail as a neuropathologist, I see ependymal cells almost every day. And I still ponder this seemingly simple epithelium.

In the past almost two centuries, much has been learned about the structure and function of ependymal cells, as well as the associated cell populations on top of and below the ependymal surface. Herein, I review the history of research concerning the ependyma and offer some perspective from the point of view of an experienced neuropathologist.

## Earliest anatomical and pathological descriptions of ependyma

**FIGURE 1:**
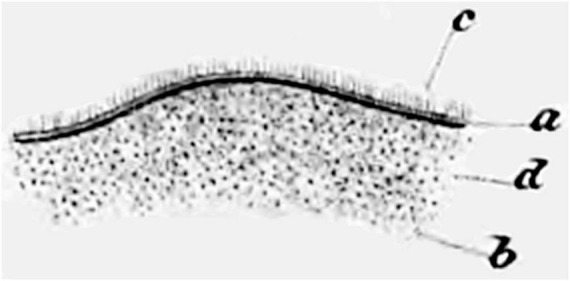
Hand-drawn image from Valentin (1836), the first to illustrate cilia on the ventricle surface. Figure 27 shows “Depiction of the fibrillation movement on the surface of the lateral ventricle of the adult human, as it is still evident during the undisturbed vibrations. a. The ciliated epithelium. b. The hairs visible as individual dots when viewed from the surface and from above. c. The upright hairs visible from the side on the folded edge. d. The balls of nerve substance that faintly shine through the ciliated epithelium” (Valentin, 1836) (illustration in public domain).

The presence of fluid-filled spaces within the brain (i.e., the ventricles) has been recognized since antiquity (Mortazavi et al., 2014). Wenzel and Wenzel (1806) wrote that the ventricles are “lined by an extremely fine membrane, which covers what little medullary substance the walls of the cavity contain, and which is sometimes easily removed from the whole inner surface when much water has accumulated in the cavity” (translated from German). In 1833, the Czech anatomist Jan Purkyně (Purkinje) and his student Gabriel Valentin were dissecting fertilized eggs from a rabbit uterus using a microscope. They observed that the surface of the oviduct was moving vigorously. Curious about it, they examined the surfaces and lumens of various organs in several species at magnifications of 300–400×. They initially stated that the “Flimmerbewegungen” (flickering movements, which were later determined to be caused by cilia) were present only on the inner surfaces of the female reproductive tract and the respiratory tract, but not on the arachnoid (Purkinje and Valentin, 1834). Two years later, Purkinje studied sheep and pig fetuses and reported that he had “finally succeeded in discovering the cilia and their movements in the entire cerebral cavities of mammals” (translated from German), albeit not on the choroid plexus epithelial cells (Purkinje and Valentin, 1835; Purkinje, 1836). He referred to these as the “Flimmermembranen” (ciliated membranes). Valentin, having moved away to establish his own laboratory, published the first illustration of ependymal cells in 1836 (Figure 1; Valentin, 1836).

The term “ependyma” (from the Greek word meaning “outer garment” or “investment”) in reference to the surface of the ventricles (the “ependyma ventriculorum”) was likely first used by the German anatomist Carl Ernst Bock a few years after Purkinje’s discovery c1839 (Šimon, 2016). Bock clearly described it as “an extremely delicate, transparent, cellular membrane, which is so intimately fused with the surface of the parts that protrude from the walls of the cerebral cavity that it can only be distinguished from them in connection with a thin, inner surface adhering layer of nerve substance” (translated from German) (Bock, 1840). The term ependyma became widely used in the anatomical literature soon after (Kolliker, 1854; Peaslee, 1957). Detailed descriptions of choroid plexus appeared during the same era (Haeckel, 1859). Some authors later advocated, albeit unsuccessfully, for use of alternate terminology such as “ependymium” (similar to “epithelium”) (Altschul, 1941).

**FIGURE 2:**
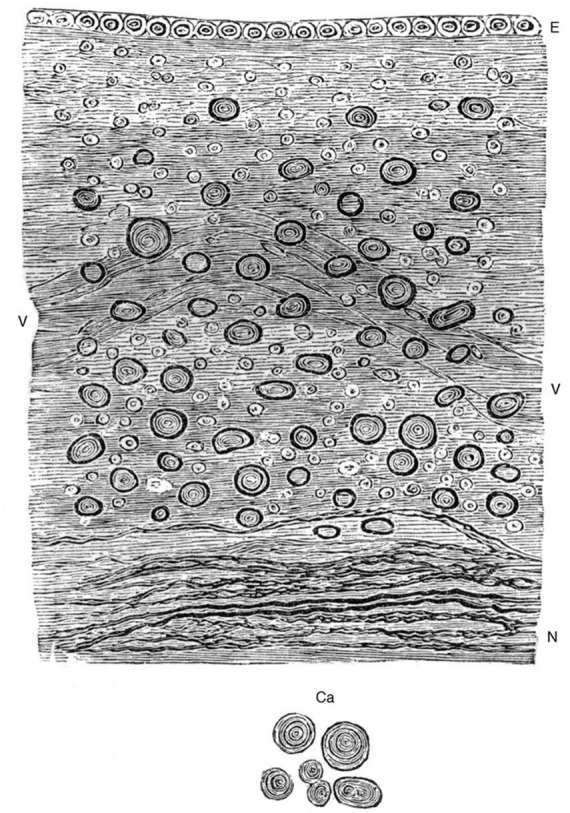
Hand-drawn image from Virchow (1858) (Figure 94) showing the relationship between the ependymal epithelium at the ventricle surface and the underlying neuroglial tissue. “Ependyma ventriculorum and neuro-glia from the floor of the fourth cerebral ventricle. E, Epithelium, N, nerve-fibers. Between them the free portion of the neuro-glia with numerous connective-tissue-corpuscles and nuclei, at V a vessel. In addition, numerous corpora amylacea, which are moreover represented separately at Ca. 300 diameters” (Virchow, 1858, 1863) (illustration in public domain).

In the mid-1800’s, the German pathologist Rudolf Virchow described brain tissues adjacent to the ependyma and introduced the term “neuro-glia” (“nerve glue”) (Virchow, 1853, 1863; Lyons, 1854). In his textbooks, Virchow suggested that the term ependyma should include the surface epithelium along with the underlying “connective tissue” (Virchow, 1863; Figure 2). It is not clear that this collective definition was widely adopted; regardless, it remains important for writers of the current literature to define what they mean by “ependyma.”

**FIGURE 3:**
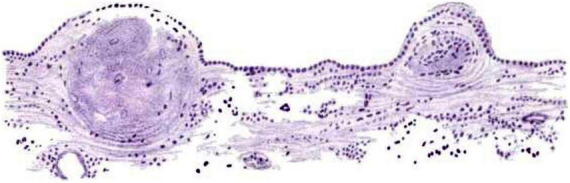
Hand-painted image from Beadles (1895) (Figure 3) showing “Ependyma of lateral ventricle from a G.P. (general paresis case) Two granulations in an early stage. The membrane is only slightly thickened, except where the nodules are appearing, and is evenly covered by a single layer of columnar-shaped epithelium. Beneath the thickenings are groups of deeply-staining cells resembling that of glandular epithelium. Logwood (stain; Haematoxylum campechianum)” (Beadles, 1895) (illustration in public domain).

Many early German pathology textbooks including (Rokitansky, 1856), and others all described granular changes along the ependymal surface in the context of hydrocephalus and infections (Walther, 1897). Friedreich (1858) described thickening and hardening of the ependymal lining following some cases of brain infection. Of particular historical interest was the perceived association between ependymal granulations (“granular ependymitis”) and insanity, syphilis, etc. (Addison, 1862; Beadles, 1895; Figure 3). Baroncini concluded in 1,888 that granulations were not specifically related to disease (Baroncini, 1888), but the concept persisted for decades (Blachford, 1903; Baird, 1910).

## Early hypotheses about the functions of the ependyma

**FIGURE 4:**
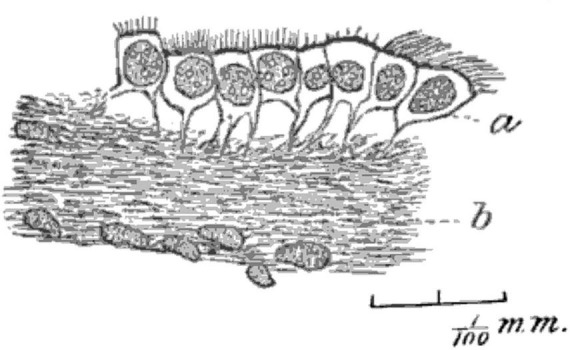
Hand-drawn image from Fish (1891) (Figure 2) used a Zeiss 8 mm objective and Abbe camera lucida. Tissue from human mesencephalon was fixed with Gaule’s method, embedded in colloidin, and sectioned. The image shows “the layer of (ependymal) cells is partially detached from the neuroglia, showing their root-like processes. Magnified about 700 diameters” (Fish, 1891) (illustration in public domain).

In the mid-1800’s, Friedreich postulated that fluid resorption occurred at the ependymal surface, with cilia facilitating this function (Friedreich, 1858). By the late 1800’s there were many hypotheses about the role(s) of ependymal cells. In 1879, Deecke published a rambling summary of suggested interactions between ependymal cells and the underlying tissues, similarities with ciliated epithelia in other organs, and possible differences between higher and lower animals. He mentioned protective, absorptive, and sensory roles (Deecke, 1879). In an 1890 study of cat brains using improved microscopy methods, Fish distinguished the ciliated nature of mural ependymal cells from the (relatively) non-ciliated choroid plexus epithelial cells. He also identified anchoring basal processes of ependymal cells in some regions of brain (Figure 4; Fish, 1891). In 1897, Eurich focused attention on the basal processes of ependymal cells and their relationship to germinal cells, which were then known as astroblasts and spongioblasts. He was one of the first to highlight the possibility that ependyma might serve a germinal function, although he was skeptical of the idea that “each individual neuroglia cell is directly derived from an ependyma cell” (Eurich, 1897).

## Improved understanding of ependymal morphology and pathology in the early 1900’s

**FIGURE 5:**
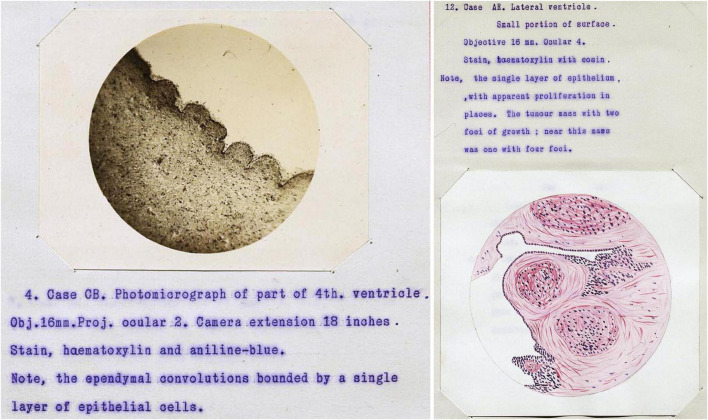
Case illustrations from Adair (1896) thesis showing the first published photomicrographs of ependymal cells (illustration in public domain).

During the 1800s, the vast majority of ependyma illustrations were hand drawn versions of microscopic observations. Likely the earliest photomicrographs of ependymal cells appeared in the 1896 thesis by Thomas Adair at Edinburgh. He published an organized review of ependyma form and function along with a detailed account of ependymal histology in a variety of species. This included the fourth ventricles of 56 humans with a range of neurological conditions including mania, melancholia (depression), dementia, syphilis, epilepsy, and hydrocephalus (Adair, 1896; Figure 5). He clearly illustrated foci of ependymal discontinuity, ependymal granulations, and buried islands of ependymal cells with and without microscopic lumens. He considered most of these to be degenerative, the result of “previous active congestions of the brain” or “acute or subacute inflammatory processes.” He also mentioned tumors “as large as a pea, or larger”; some of these likely represent subependymomas (see section titled “Neoplasms of the ependyma”).

**FIGURE 6:**
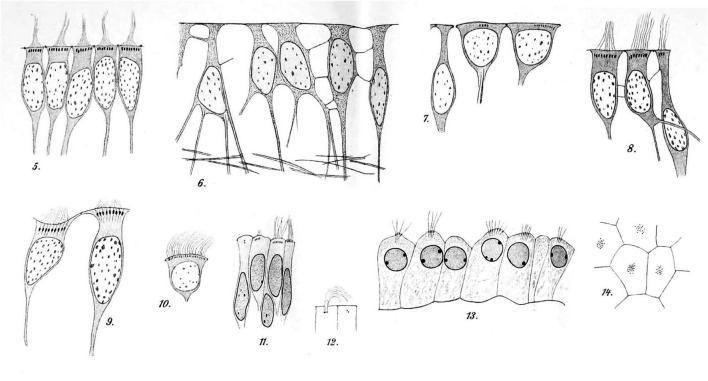
Hand-drawn images of ependymal cells from human brains [reproduced and re-organized from Studnička (1900) Tafel XXXII/XXXIII]. Images 5–10 are ependymal cells of the fourth ventricle of an adult. Images 11 and 12 are ependymal cells from the lateral ventricle of a fetal brain. The dark spots at the base of the cilia are the basal bodies. Images 13 (sectioned) and 14 (en face) show epithelial cells of the lateral ventricle choroid plexus from a fetal brain. Tissues had been processed with Muller’s fixative (potassium dichromate), sectioned, and stained with iron hematoxylin (Studnička, 1900) (illustration in public domain).

In 1900, František Studnička from Prague published a beautifully detailed account (with drawings only) summarizing prior work and describing regional anatomical differences in the ependyma of species ranging from lower vertebrates to humans. He wrote “the ependyma is present everywhere on the inner surface of the cerebrospinal tubes” and he described situations of secondary loss with transformation into neuroglial cells. He demonstrated at the base of each cilium small “Basalkörperchen” (basal bodies) or “Blepharoplasten” (blepharoplasts) (Figure 6; Studnička, 1900).

During the decades soon after, there was little progress. Anatomists and pathologists continued to describe areas of ependyma loss in varied circumstances and the associated proliferation of glial tissue along the ventricle walls (“ependymal gliomatosis”) (Margulis, 1913). Serial sectioning of tissue showed that some clusters of ependymal cells below the ventricular surface were truly isolated “rosettes” while others represented parts of narrow diverticulae. Incorrectly, some of these were believed to be the result of ependymal cell hyperplasia (Altschul, 1941).

Increasingly in the 1930’s, publications concerning ependymal biology and pathology were written in English rather than German. It was becoming clear that some infectious agents (e.g., syphilis and tuberculosis) could damage the ependymal lining of the ventricles thereby scarring the CSF pathways and causing hydrocephalus (Arnold, 1934). Several investigators evaluated acute ependymal changes following blunt head trauma. Rand and Courville (1931) reported vacuolization of ependymal and choroid plexus epithelium in association with brain edema. Gierlich (1936) documented “hemorrhages under intact and defective ependyma” in many autopsy cases. Most textbooks of anatomy had fairly superficial contemplations of ependymal cells, considering them to be a terminally differentiated cell population (Ranson, 1935). However, in one textbook of neuropathology, the author suggested many possible roles of ependymal cells (a) in support of subjacent cells, (b) as a possible source of fluid secretion including CSF and specialized protein secretions such as from the subcommissural organ (to produce Reissner’s fiber), (c) as an organ for sensing CSF composition especially via associated nerve fibers, and (d) as an organ of generation and regeneration. These were being considered particularly likely in lower vertebrates but conceivably also in man (Agduhr, 1932). Around the same time, physiologic studies on living animals were used to study CSF production by choroid plexus (Putnam and Ask-Upmark, 1934).

## Central canal of the spinal cord

From the earliest descriptions, ependymal cells similar to those in the ventricles were known to line the central canal of the spinal cord (Virchow, 1854; Deecke, 1879). Generally, they were considered as a continuum, but some differences were noted. Proximity to a proteinaceous structure within the central canal was observed in the mid 1800s (Reissner, 1860; Sanders, 1878). This came to be known as the Reissner fiber, which originates as a secretion from the subcommissural organ at the cranial end of the cerebral aqueduct (Aboitiz and Montiel, 2021). Around 1900, interest grew in the proliferative capacity of spinal cord ependymal cells and/or closely related germinal cell populations. Hamilton described mitoses among ciliated ependymal cells in the lumbar cord of postnatal rats (Hamilton, 1901). Hooker (1915) speculated that ependymal cells play a role in frog spinal cord regeneration. However, it was not until the 1950s that interest and experimentation on this relationship in fish, amphibians, and reptiles expanded (Piatt, 1955; Anderson and Waxman, 1983). The extent to which ependymal/progenitor cells can contribute to recovery from spinal cord damage in mammals is the subject of recent research and is beyond the scope of this article (Marichal et al., 2017).

Streeter described regression of the ependymal cell layer during embryonic development of the human filum terminale (Streeter, 1919). Schultze (1882) provided histopathologic descriptions of syringomyelia (enlargement and disruption of the central canal) and obliteration of the central canal by ependymal cells. The latter situation was eventually recognized as a near unanimous state in adult humans (see section titled “Ependymal cell loss”).

## Study of ependymal cells by electron microscopy

**FIGURE 7:**
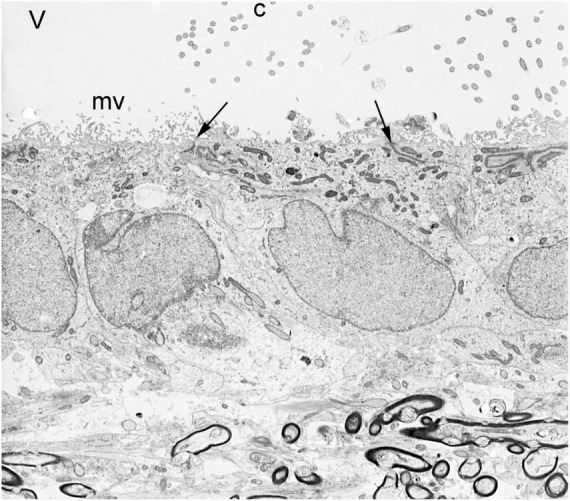
Transmission electron micrograph of normal ependymal cells lining the lateral ventricle of an adult rabbit brain. V = ventricle, c = cilia, mv = microvilli, arrows = apical junctional complexes, dark structures at bottom = myelinated axons of corpus callosum. Magnification 4,300×.

Owing to the limits of photon wavelengths, spatial resolution of light microscopy in the 20th century (up to 1,000× magnification) could not reveal much beyond the epithelial and ciliated nature of ependymal cells. Electron microscopy opened new avenues of research. Transmission electron microscopy (TEM), which uses electrons rather than photons, allows > 100,000× magnification. However, the techniques for preparation of biological samples were crude in the early 1950’s. TEM was initially used to demonstrate the “brush border” (microvilli) on mouse choroid plexus epithelial cells, which were easy to isolate. Microvilli were thought to be an absorptive organelle, so the investigators did not understand their role on secretory cells (Dalton et al., 1950, 1951). By the mid-1950’s, TEM methods had improved considerably and were increasingly used by numerous research groups for detailed studies of choroid plexus epithelial cells (Maxwell and Pease, 1956) and the demonstrations of microvilli, intercellular junctions, and basal lamina on ependymal cells (Luse, 1956; Pease, 1956; Brightman and Palay, 1963; Klinkerfuss, 1964; Malinsky, 1968; Leonhardt, 1972; Figure 7). Injection of ferritin particles into the CSF compartment showed that the ependymal layer serves at least a partial barrier to entry of molecules into brain parenchyma (Brightman, 1965).

**FIGURE 8:**
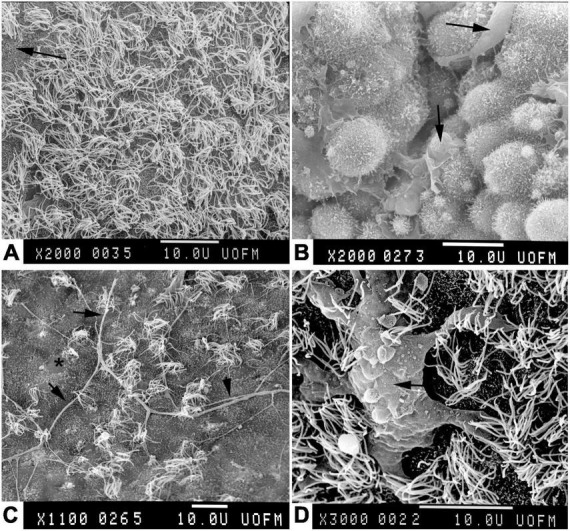
Scanning electron micrographs of adult rabbit brains. **(A)** Normal ventricle wall with normal density of apical cilia on ependyma. Where cilia are sparse, microvilli can be seen (arrow). **(B)** Normal choroid plexus epithelial cells with apical macrophages (Kolmer cells, arrows). Note the paucity of cilia in comparison to ependymal cells. **(C)** Ventricle wall of rabbit that had been hydrocephalic for 4 weeks. Note the sparsity of ependymal cilia, focal loss of microvilli (*), and the long supraependymal axon processes (arrows) (Del Bigio, 1987). **(D)** Supraependymal macrophage (arrow) with blunt processes on the ependymal surface of a rabbit that had an intraventricular implant for 3 days (Del Bigio and Bruni, 1986).

Transmission electron microscopy was typically used to study ependyma in a plane perpendicular to the ventricle surface, clearly showing how ependymal cells are organized with respect to their neighbors in the underlying glial layer. However, understanding the overall organization of the ventricle surface required some guess work. Uncertainty was mitigated by scanning electron microscopy (SEM), which conveys information about surface topology. Initially in 1965 SEM was applied to human lung tissue samples with suboptimal results (Jaques et al., 1965), but techniques improved rapidly. By the early to mid 1970’s SEM was used for elegant studies of the inner brain surfaces of large animals including humans (Bruni et al., 1972; Clementi and Marini, 1972; Noack et al., 1972; Scott et al., 1972; Weindl and Joynt, 1972; Allen and Low, 1973). Detailed studies of cilia distribution in normal and pathological conditions (especially hydrocephalus wherein the ventricles are enlarged and their surfaces stretched) were conducted (Borit and Sidman, 1972; Nielsen and Gauger, 1974; Page, 1975). The combined approaches of TEM and SEM allowed detailed investigations of the specialized circumventricular organs (Weindl, 1965), especially around the third and fourth ventricles (Fleischhauer, 1961; Bruni, 1974). Modern publications substantiate the utility of electron microscopy in the discrimination of subtle regional differences in these ependymal populations (Lorencova et al., 2020). Furthermore, SEM revealed the full extent of supraependymal axons and macrophages (Mitchell and Ham, 1998; Figure 8) (see below). SEM was frequently used for characterization of ventricular surfaces for about three decades, but since 2000 it has been used in only a few publications per year for very focused applications.

## Ependymal cells *in vitro*

Hogue (1947) cultured explants of cerebellum from a 90 mm human fetus (approximately 15 weeks gestation) and observed that ependymal cilia remained active for at least 90 days *in vitro*. A decade later in separate labs, Friede and Hild grew ependymal cells in monolayers thereby allowing direct observation of cilia activity under a range of physiologic and pharmacologic conditions (Friede, 1955; Hild, 1957). However, it was not until the 1960’s onward that *in vitro* experiments became more frequently used for interrogation of ependymal cell properties including the developmental biology, permeability, and mechanics of cilia motion (Hild et al., 1965; Singer and Goodman, 1966; Dalen et al., 1971; Abney et al., 1981; Laabich et al., 1989; Delgehyr et al., 2015). Co-culture experiments showed that an underlying layer of astrocytes was important for differentiation of ependymal cells (Araki et al., 1983).

## Role of ependymal cilia

As described in detail above, cilia were identified as the distinguishing feature of ependymal cells lining the ventricles of the brain. Speculation about their function began immediately. Purkinje and others assumed that ependymal cilia serve to move fluid or debris, much as in the airway (Purkinje, 1836). However, it was a century before direct hypothesis testing became possible. Work in the 1960’s showed coordinated cilia activity on explanted brain fragments indicating that they might create currents in CSF at least in proximity to the brain surface (Worthington and Cathcart, 1963; Dalen et al., 1971). In live frogs and rats, ependymal cilia were shown to move in the general anatomical direction of CSF flow and could rapidly move cell debris (Cathcart and Worthington, 1964; Worthington and Cathcart, 1966; Nelson and Wright, 1974). However, direct comparison with airway tissue showed that ependymal cilia beat more rapidly (about 30 cycles per second) are less efficient particle carriers (Roth et al., 1985). Recent work confirms the cilia beat gradients described decades earlier, but show that they establish local flow networks to preferentially distribute small molecules and that they do not contribute to bulk flow of CSF (Faubel et al., 2016). TEM was the mainstay for studying cilia structure for decades (Roy et al., 1974), but immunohistochemistry and immunofluorescence are now equally or more valuable through their ability to localize specific protein components.

Even in the absence of overt pathology, almost all ependymal cells are immunoreactive for ubiquitin (Kawanishi et al., 2003), which is a protein involved in degradation and recycling of proteins through the proteasome. This was initially thought to indicate that ependymal cells are subject to proteotoxic stresses not encountered by other brain cells (Kandel et al., 2023). However, the more likely explanation is that motile cilia proteins along with Foxj1 transcription factor have high turnover rates that require ubiquitin for recycling (Stephens, 1999; Long et al., 2015; Abdi et al., 2018; Hao et al., 2021).

## Axons and nerves associated with the ependymal layer

Silver impregnation was used to label axons running alongside subependymal blood vessels of the human ventricular system in the 1930’s. Such axons have scattered projections into the ependymal layer (Deery, 1935; Frey and Stoll, 1947). These CSF-contacting neurons were studied extensively by Vigh and Vigh-Teichmann (1971, 1998) beginning in the 1960’s. Most are situated in the spinal cord, fourth ventricle, and third ventricle where, current data show, they serve as chemoreceptors (Wyart et al., 2023).

An apparently distinct set of intraventricular (supraependymal) axons runs on top of the ependymal layer (Figure 8). Beginning in the mid 1960’s Leonhardt and Lindner (1967) and Leonhardt and Lindemann (1973) as well as others conducted extensive morphological studies of these axons in experimental mammals (Alvarez-Morujo et al., 1992). During the 1970’s in rat experiments, they were shown by autoradiography and later by immunohistochemistry to originate in the raphe nucleus of the brainstem. These axons were postulated to release serotonin directly into the CSF (Aghajanian and Gallager, 1975; Chan-Palay, 1976; Tramu et al., 1983), but in 1979 synaptic contacts between serotoninergic axons and ependymal cells of the rat subcommissural organ were demonstrated (Mollgard and Wiklund, 1979). Soon after, supraependymal axons were demonstrated in the human brain (Richards et al., 1980). Interest in the direct effects of serotonin on ependymal cells began in the 1960s (Svanidze and Bregvadze, 1968; Jorge, 1977). Lorez (1976) hypothesized that serotonin regulates cilia activity; however, direct experimentation on the exposed rat fourth ventricle failed to prove so. Although previously shown in other cell types, it was not until 25 years later in 2001 that serotonergic stimulation of ependymal cilia beating frequency was shown directly in cell culture (Nguyen et al., 2001) (see section titled “Role of ependymal cilia”). Recent work suggests that supraependymal axons might also contribute to regulation of the subependymal stem cell niche (Tong et al., 2014).

## Supraependymal macrophages

Microglial cells, which were first described c1920 by del Río-Hortega (1919, 1920), are the resident macrophage/immune cell population of the central nervous system. Distinct populations have been identified diffusely within the brain parenchyma, in perivascular compartments, and in the meninges. A discrete subependymal population was identified in macaque monkeys in 1937 (Dewulf, 1937) and described in great detail in rabbits in 1965 (Cammermeyer, 1965). SEM studies in the 1970’s showed a widespread population of cells adherent to the ventricle surface (Coates, 1973). Structural and functional studies showed that these too are a resident monocyte/macrophage population (Albrecht and Bleier, 1979) similar to (Kolmer) epiplexus cells that are located on the surfaces of choroid plexus epithelial cells (Allen, 1975; Schwarze, 1975; Figure 8).

In the 1980’s, experimental injections of peptides or induced inflammation (injection of bacillus Calmette-Guerin into cisterna magna) showed that the supraependymal cells originated from the periventricular blood vessels and could increase in quantity by enhanced extravasation or by local proliferation (Merchant and Merchant, 1980a,b; Saland et al., 1983, 1984). As elsewhere, the main role of these cells appears to be phagocytosis in response to periventricular cell injury, infection, or hemorrhage.

## Ependymal cell loss

**FIGURE 9:**
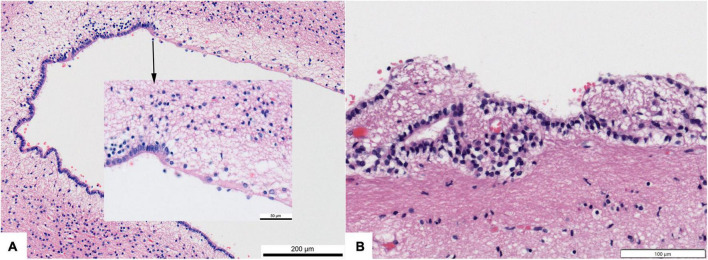
Photomicrographs showing regional loss of ependyma in otherwise normal human brains. **(A)** Dorsolateral angle of frontal horn of lateral ventricle in a 4 month infant (200× original magnification). Inset–transition to ependyma loss (600×). **(B)** Focal loss and buried islands of ependymal cells along the third ventricle of a 68 year old. All hematoxylin and eosin stain.

“Accidental” loss of ependymal cells was recognized as a common feature of human brains in the 1930’s (Agduhr, 1932). Johnson and Johnson (1972) reported that 65/100 adult brains had focal loss of ependyma from the lateral ventricle walls; in association there was “increased accumulation of glial fibers or loosening of glial fibers, or both.” There was no difference between young adults and the elderly “suggesting that the lesion dated from preadult life” (Johnson and Johnson, 1972). Dooling et al. (1977) studied the ependymal lining of 111 human fetuses. They showed that focal loss of ependyma becomes apparent in occipital horns of the lateral ventricles as early as 23 weeks gestation, several weeks after the germinal matrix involutes and ciliated ependymal cells first appear. Patchy loss of ependyma along the corpus callosum and in the temporal horn over the hippocampus appears by 25 weeks and progresses with advancing gestation (Dooling et al., 1977). My personal experience with 1000’s of brains is that gaps in the ependyma are ubiquitous (while perhaps not “normal”) along the human callosum and occipital horns and that they tend to become more widespread in the lateral ventricles with increasing age (Figure 9). Accompanying sites of ependyma loss are increases in the thickness of the periventricular astroglial layer. Experimental studies in rats showed that subependymal germinal cells proliferate and differentiate into astrocytes in this situation (Willis et al., 1976). In his excellent 1995 review paper, Sarnat addressed the matter of subependymal or subventricular gliosis extensively (Sarnat, 1995).

In the lateral ventricles of humans, patchy ependyma loss seems to be a benign phenomenon. Less often in association with developmental anomalies, regional adhesion of lateral ventricle walls occurs where ependymal cells are absent (Davidoff, 1946). In rodents, the apical surfaces of otherwise intact ependymal cells can become entangled such that the ventricular surfaces fuse (Sturrock, 1979).

Ependymal disruption and disorganization in the spinal cord is accompanied by obliteration of the central canal in almost all humans by the third decade (Agduhr, 1932; Kasantikul et al., 1979; Milhorat et al., 1994; Torrillas de la Cal et al., 2021).

Of greatest clinical significance is ependymal disorganization along the cerebral aqueduct. The aqueduct has a continuous ependymal lining in essentially all normal human brains, although there may be small diverticulae (Emery and Staschak, 1972; Alvarez et al., 1987). So-called “stenosis” of the cerebral aqueduct, which is potentially associated with severe enlargement of the lateral ventricles (hydrocephalus), was known to be associated with a disrupted ependymal cell layer by 1900 (Del Bigio, 1993). Integrity of the ependymal layer is necessary for maintenance of aqueduct patency in rodents (Paez et al., 2007) and ependyma loss followed by wall adhesion and glial “scarring” may be the cause of aqueduct narrowing (Del Bigio, 2014).

The cause of ependymal cell loss is not known with certainty in individual human cases, but compelling epidemiological and experimental evidence indicates that otherwise minor viral infections might be responsible. Beginning in the 1940’s, inoculation of experimental chicks and mice with influenza virus was shown to infect and destroy ependymal cells (Henle and Henle, 1946). Ependymal cells are susceptible to several other common viruses including mumps (Hamburger and Habel, 1947; Johnson et al., 1967) and parainfluenza (Mims and Murphy, 1973; Tardieu and Weiner, 1982). These viruses do not have a direct impact on other glial cell populations or neurons. Experimental reovirus infection in hamsters causes necrotizing ependymitis and obliteration of the spinal central canal (Milhorat and Kotzen, 1994). Since c1900 it has been known that degenerative ependymal changes also occur as a consequence of hydrocephalus during which stretched ependymal cells become flattened and cilia regress (Del Bigio, 1993). If ventriculomegaly occurs in the fetus, loss of ependyma coverage appears to compromise the underlying subventricular zone (Dominguez-Pinos et al., 2005) (see section titled “The relationship of ependymal cells to the brain’s germinal cell population”).

Gaps in the ependyma likely persist because mature ciliated cells do not readily proliferate following injury, as has been shown in rat brains (Bruni et al., 1983) and human spinal cord (Paniagua-Torija et al., 2018).

## Barrier function

The concept of blood-brain, brain-CSF, and external meningeal physiologic barriers arose early in the twentieth century. Decades later in the 1960’s it became apparent from ventricular infusion studies that an intact ependymal layer could serve as a partial barrier capable of blocking passive movement of large molecules from the CSF into the brain parenchyma (Pollay and Davson, 1963; Pollay, 1967; Rall, 1968; Del Bigio, 1995b). Small molecules, ions (e.g., K^+^), and water move freely across the ependyma (Cserr, 1965). Robust barrier function would not be expected because ependymal cells are attached to each other by zonula adherens type junctions and not tight junctions (Brightman and Reese, 1969). It should be noted that the inter-ependymal junctional complexes are more complex in immature sheep brains when germinal matrix cells are still proliferating (Mollgard et al., 1987).

Many experiments with large animals in the 1970’s indicated that ventricle wall permeability to tracers is increased in association with hydrocephalus (Milhorat et al., 1970). The ependymal layer is attenuated and disrupted when the ventricles enlarge (Del Bigio, 1993). Considering that ventricular outflow of CSF is reduced, it is not clear if tracer flux changes indicate a true increase in permeability or more simply an altered fluid equilibrium.

## Relationship of ependymal cells to the brain’s germinal cell population

It has been known for decades that ciliated ependymal cells differentiate at the ventricular surface as proliferation in the adjacent subependymal zone declines (Del Bigio, 2011). Studies of immature animals in the 1960’s showed that the periventricular germinal tissue begins as a poorly differentiated stratified cell layer and matures into a simple ciliated epithelium (Tennyson and Pappas, 1962). The biology of this transformation is understood only through very recent work (i.e., <20 years) and readers are referred to other sources (cited here). Briefly, several signaling mechanisms drive terminal differentiation of ependymal cells (Spassky et al., 2005; Kyrousi et al., 2017; Redmond et al., 2019; Stratton et al., 2019). Other writers address the complex debate about whether ciliated ependymal cells are themselves progenitors or if they act only in a supporting or protective role for the subependymal cell progenitor population (Stensaas and Gilson, 1972; Ohata and Alvarez-Buylla, 2016; Coletti et al., 2018; Shah et al., 2018).

## Neoplasms of the ependyma (ependymoma and subependymoma)

The term “ependymoma” appeared c1898 (Benda, 1898) although periventricular brain tumors had been previously described (Mallory, 1902). Bailey cautioned in 1924 that most tumors called “ependymal gliomas” likely did not originate from ependymal cells and merely were located near the cerebral ventricles. He stated that “true ependymomas” should be “tumors originating from ependymal cells and more or less closely reproducing their structure” (Bailey, 1924). The classic ependymoma recapitulates some of the features of ependymal cells including intercellular junctions and cilia projecting into microlumens. Myxopapillary ependymoma, a morphologically distinct variant that arises from the filum terminale of the spinal cord, was identified in 1933 (Kernohan et al., 1933). A benign variant of periventricular ependymoma, the subependymoma, was recognized in 1945 (Scheinker, 1945). Dorothy Russell, one of the doyens of tumor neuropathology, called it a “dubious addition” (Russell, 1950) but it remains a legitimate entity in the classification of brain tumors (Louis et al., 2021). Knowledge of the genetics of ependymoma and possible origins from radial glial began to emerge in the mid 2000s (de Bont et al., 2008; Mack and Taylor, 2017) and have advanced rapidly since (Junger et al., 2021; Santi et al., 2022).

## Summary and comments regarding relevance to human neuropathology

Research during the past two centuries had shed considerable understanding on the role of the ependyma, a simple and often ignored cell population in the brain. Neuropathologists and neurobiologists have published numerous good reviews concerning the ependymal structure and function in past decades (Bargmann et al., 1982; Bruni et al., 1985; Sarnat, 1992, 1995, 1998; Del Bigio, 1995a,b, 2010; Bruni, 1998; Moore, 2016; Nelles and Hazrati, 2022; Deng et al., 2023). Other reviews appear as chapters in books whose major subject is hydrocephalus (Vidovic et al., 2016). Readers are referred to these papers for more in depth considerations of some of the intermediate history.

In my work as a neuropathologist who examines human brains across the full age spectrum, what have I learned about the ependyma in normal brains? Usually it is present. Some regions, for example over the corpus callosum or in the occipital horns of the lateral ventricles, exhibit foci of ependymal attenuation or loss. These foci often have minor, focal chronic astroglial hyperplasia or buried islands of ependymal cells. In the circumstance of infection (e.g., ventriculitis) or intraventricular hemorrhage the ependymal layer is overtly and often widely disrupted. This can have serious consequences if the cerebral aqueduct becomes scarred, leading to hydrocephalus which, in turn, further disrupts the ependyma.

The enduring questions I have about ependyma in the context of clinical neuropathology are the following:

(1) Considering their relationship to periventricular germinal cells, are ependymal cells critical during brain development and merely vestigial in the adult?
(2) If gaps in ependymal coverage are ubiquitous, does this create a weakness in a brain barrier that should be targeted for intervention, or does it mean that in some regions of the ventricular system that these cells are not necessary after a certain developmental stage? Furthermore, to what extent does the thickened periventricular astroglial layer replace the function of the ependyma?
(3) During human development (or in the brains of small animals) do ependymal cilia serve to keep narrow CSF channels (i.e., the cerebral aqueduct) clear of debris, which might act as a nidus for scarring? Why does ciliary dyskinesia cause hydrocephalus in so many murine genetic disease models but in so few humans (Sakamoto et al., 2021)? To what extent are ependymal cilia critical in mature humans for microdistribution of small molecules within the ventricular system?
(4) Do we pay enough attention to “minor” viral illnesses and their possible effects on the brain?
(5) Do we understand enough about the resident supraependymal macrophage population and its responses to intraventricular hemorrhage or infection? In the context of hydrocephalus, does this population contribute to the foreign body type inflammatory reaction that is associated with surgical shunt systems used to treat hydrocephalus (Del Bigio, 1998)?

Finally, reflecting back on Virchow’s suggestion that the term ependyma should include the surface epithelium along with the underlying “connective tissue” (Virchow, 1863). I must disagree. Unless we clearly define the cell populations we talk and write about, subsequent generations of researchers might be confused unless they read the primary work in detail. Please reserve the term “ependyma” for the mature, simple ciliated epithelium of the central nervous system.

## Author contributions

MD: Conceptualization, Writing – original draft, Writing – review and editing.
